# Effect of Roasting Time and Cryogenic Milling on the Physicochemical Characteristics of Dried Ginseng Powder

**DOI:** 10.3390/foods9020223

**Published:** 2020-02-20

**Authors:** Hayeong Jeong, Dong Hyeon Park, Han Geuk Seo, Mi-Jung Choi, Youngjae Cho

**Affiliations:** Department of Food Science and Biotechnology of Animal Resources, Konkuk University, 120 Neungdong-ro, Gwangjin-gu, Seoul 05029, Korea; twinhy2@naver.com (H.J.); doom11@naver.com (D.H.P.); hgseo@konkuk.ac.kr (H.G.S.); choimj@konkuk.ac.kr (M.-J.C.)

**Keywords:** cryogenic milling, ginseng, particle size, physiological activity, roasting, water solubility

## Abstract

This study aimed to evaluate the effect of reduced particle size of ginseng by roasting and cryogenic milling on increasing its water solubility and physiological activity. The samples were roasted for different times (9–21 min) and generated in different sizes (10–50, and >50 μm). All roasted samples revealed significantly smaller particle sizes than did non-roasted samples, based on Sauter mean diameter (D [3,2], *p* < 0.05). Furthermore, the particle sizes of roasted samples decreased until roasting up to 15 min. In terms of the water solubility index (WSI), antioxidant activity, total polyphenol content (TPC), and total polysaccharides according to particle size, 10–20 μm-sized samples showed the highest values when compared with >50 μm-sized samples. Based on roasting time, WSI values of all samples roasted for up to 15 min were higher than those of the control (not roasted) (*p* < 0.05). Antioxidant activity and TPC also increased with increasing roasting time. Total polysaccharide content was the highest upon roasting for 15 min, except for the 10–20 μm sample. Ginsenoside content of roasted samples >20 μm size was higher than that of the control (not roasted) except after 15 min of roasting. Therefore, roasting and cryogenic milling are effective in producing ginseng root powder.

## 1. Introduction

Ginseng (*Panax ginseng* C.A. Meyer) has been known as general herbal medicine and functional food in south and southeast Asia for over 2000 years [1]. Ginseng root is more widely used than the leaf and stem of ginseng because of its lower residual pesticides [2]. Ginseng roots also have many bioactive constituents, such as ginsenosides, acidic polysaccharides, and phenolic compounds [3,4,5]. In particular, major ginsenosides (Rg1, Re, Rf, Rb1, Rc, Rb2, Rb3, and Rd) and minor ones (Rg2(S), Rg2(R), Rg3(S), Rg3(R), and Rh1(S)) in ginseng roots have many functions, including anti-cancer, antioxidation, anti-stress, blood pressure adjustment, and immune system enhancement effects [6,7,8]. Currently, ginseng products mostly exist in the form of extracts and powders [9]. Ginseng powder forms are more effective than extract forms, because powder forms contain all the bioactive components with hydrophilic, hydrophobic, and amphiphilic properties [9]. However, ginseng powder has problems of low water solubility, which need to be improved.

Roasting is an appropriate pretreatment solution to improve the low solubility of food products. When roasting raw materials, various components are changed by reactions such as decomposition, synthesis, and condensation [10]. This facilitates the dissolution of components contained in food by high temperature [10]. Furthermore, the roasting treatment breaks down the plant cell wall and increases intracellular space, resulting in easy extraction of bioactive components and increased water-soluble contents [11,12,13]. Thus, roasting increases the ginsenoside, browning substance (melanoid), acidic polysaccharide, and antioxidant contents [14,15,16,17]. Along with roasting, this study uses a cryogenic (very low temperature) milling machine to prepare ginseng powder, which is not common in commercial ginseng grinding. Cryogenic milling is a pulverization method that uses a liquid nitrogen bath at −196 °C.

Cryogenic milling reduces particle size by weakening the cell wall and increases the water solubility index when compared with effects of high temperature milling [18]. Manohar et al. (2001) reported that the particle size of *Curcuma domestica* subjected to cryogenic milling (8.56 μm) was smaller when compared with effects of hammer milling (88.20 μm) [19]. Cryogenic milling also prevents discoloration and oxidation by high heat [20].

Therefore, this study aims to investigate the effect of roasting and cryogenic milling on increasing the water solubility and bioactive constituents in ginseng root. Experiments are also performed to compare the physicochemical properties, such as particle size, appearance, color, water solubility index (WSI), DPPH scavenging activity, total polyphenol content (TPC), total/acidic polysaccharide content, and ginsenoside content, based on particle size and roasting.

## 2. Materials and Methods

### 2.1. Ginseng Preparation and Pretreatment

Four-year-old dried ginseng roots (*Panax ginseng*) were purchased from Bio hongsam (Geumsan, Korea). Ginseng roots were cut to approximately 0.5 cm thickness and the cut ginseng roots (125 g) were roasted for 9, 12, 15, 18, and 21 min at 180 °C using a roaster (CBR-101; Gene Café, Ansan, Korea).

### 2.2. Preparation of Roasted Ginseng Powder

Roasted ginseng roots were ground using a cryogenic milling machine (SPEX 6875D Freezer/Mill; Spex sampleprep, Metuchen, NJ, USA). The milling machine was pre-cooled for 5 min to reach −196 °C before grinding. The ginseng was placed in a vial and milled at 15 cycles per second (CPS, the number of cycles in which the steel impactor moved forward and backward per second) for 3 min. The milling machine was also cooled for 1 min between repetitions of the milling cycle.

### 2.3. Treatments

**Figure 1:**
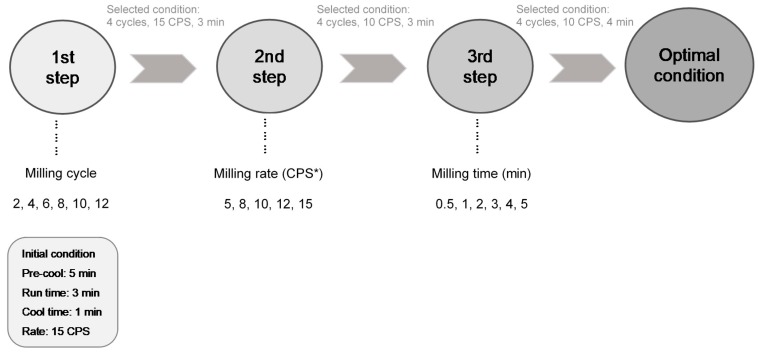
Cryogenic milling conditions for dried ginseng root. The condition of a cycle was 3 min milling at a rate of 15 CPS (cycles per second). CPS* indicates the number of back-and-forth cycles per second completed by the impactor.

Figure 1 presents the cryogenic milling conditions of dried ginseng root. The cryogenic milling condition was set in three stages, including the cycles, rate (CPS), and time. The conditions with a cycle were based on 5 min of precooling, 3 min of milling time, 1 min of cooling time, and 15 CPS milling rate. The ranges of each stage were set to 2–12 cycles, 5–15 CPSs, and 0.5–5 min. After cryogenic milling of ginseng treatments under different conditions, the treatments were classified according to particle size based on the Sauter mean diameter (defined as the diameter of a sphere that has the same volume/surface area ratio as a particle of interest), D [3,2]. In the range of each size, one treatment was selected as a representative condition. The samples were roasted and then ground by cryogenic milling using the selected condition. Roasting as a pretreatment step was performed for 9, 12, 15, 18, and 21 min at 180 °C.

### 2.4. Particle Size

Particle size was measured using a laser diffraction particle size analyzer (Mastersizer 3000; Malvern, Worcestershire, UK). The measurement conditions of particle size were presented in a dry condition as follows: refractive index of particles (a number that describes how light propagates through the particles), 1.520; absorption index of particles, 0.01; dispersion medium, dry; dispersion refractive index, 1; particle shape, non-spherical. After placing the ginseng powder on the specimen inlet, the sample was automatically injected using the flow rate (50% power) and the feed rate (50% power). The particle size of ginseng powders was analyzed five times.

### 2.5. Color

A Petri dish with a size of 35 × 10 mm was completely filled with ginseng powders. Color parameters of ginseng powder were measured using a CR-400 colorimeter (Konica minolta sensing, Tokyo, Japan) after calibration with a standard plate (L*, 96.67; a*, 0.21; and b*, 1.82). All samples were measured five times. The total color difference of ginseng powder was calculated using the following equation:

(1) $$\Delta E\;=\;\sqrt{\Delta L^{2}+\Delta a^{2}+\Delta b^{2}}.$$

### 2.6. WSI

The average WSI of all ginseng powders was analyzed in triplicate, as described by Lee et al. (2013) [21]. Ginseng powder (0.5 g) was dispersed in 30 mL of distilled water, and the suspension (water soluble and insoluble ginseng contents) was shaken for 60 min at 80 °C in a water bath (BF-30SB; Biofree, Seoul, Korea). The suspension was then centrifuged using a centrifugal separator (1736R; GYROZEN, Daejeon, Korea) for 30 min at 9200× *g*. The supernatant was filtered through a Whatman No. 2 paper (Sigma, St. Louis, MO, USA). Next, the filtered solution was dried for 24 h at 60 °C using a hot air dryer (LD-918TH; Lequip, Hawsung, Korea). WSI was calculated using the following equation:

WSI (%) = (weight of dried ginseng solution/weight of ginseng powder) × 100.(2)

### 2.7. DPPH Free Radical Scavenging Activity

Antioxidant activity was measured by modifying the Blois method of removing 2,2-diphenyl-1-picryl-hydrazyl (DPPH) (Sigma, St. Louis, MO, USA) free radical, which is a stable nitrogen-centered radical [22]. Each 1 g of ginseng sample was extracted with 25 mL of 70% ethanol using a water bath (BF-30SB; Biofree) for 3 h at 80 °C. The extracted ginseng suspension was filtered through a Whatman No. 2 paper (Sigma) and the filtrate was concentrated using an EYELA rotary evaporator N-1000 (Sunileyela, Seongnam, Korea). The concentrate was then lyophilized using a freeze-dryer (MCFD8512; Ilshinbiobase, Yangju, Korea). The concentrate (160 μL) was diluted with 99% methanol, and different concentrations of the diluted solutions (5, 2.5, 1.25, 0.625, and 0.3125 mg/mL) were mixed with 40 μL of DPPH reagent (Ab 0.95–0.99). In the same way, instead of DPPH reagent, methanol (40 μL) was added to the ginseng solution (160 μL) for the calibration of the sample color. The control was prepared by mixing methanol (160 μL) and DPPH (40 μL). All the samples were reacted at room temperature (22–24 °C) in the dark for 10 min. The absorbance of the samples was analyzed at 517 nm using a Multiskan^TM^ GO plate reader (Thermo Scientific, Waltham, MA, USA). The scavenging activity of DPPH free radicals was calculated by the following equation:

DPPH free radical scavenging activity (%) = (1 – ((A1 − A2)/A3)) × 100,(3)

where A1 is the absorbance of the DPPH solution (40 μL) + ginseng solution (160 μL), A2 is the absorbance of the methanol (40 μL) + ginseng solution (160 μL), and A3 is the absorbance of DPPH (40 μL) + methanol (160 μL).

Next, the antioxidant activity was converted to an half maximal inhibitory concentration (IC50) value representing the amount of ginseng that removed 50% of DPPH free radical.

### 2.8. Polyphenolic and Polysaccharide Extract Preparation

To manufacture ginseng extract, 0.5 g of ginseng powder was dispersed in distilled water (50 mL) using a water bath (BF-30SB; Biofree) for 2 h at 95 °C. Ginseng suspension was then centrifuged using a centrifugal separator (1736R; GYROZEN) for 10 min at 9200× *g* and filtered. Then, samples were adjusted to a volumetric flask (50 mL) for the measurement of physicochemical characteristics (the TPC and the total and acidic polysaccharide contents).

#### 2.8.1. TPC

TPC was measured using the Folin–Ciocalteu method (Ough and Amerine 1980) [23]. Ginseng extract (2 mL) was vortexed with 2 mL of Folin–Ciocalteu reagent for 3 min, and 10% Na_2_CO_3_ (2 mL) was added to the mixture. The final mixtures were reacted for 1 h at room temperature in the dark, and the sample absorbance was analyzed at 700 nm using a Multiskan^TM^ GO plate reader (Thermo Scientific). The TPC was calculated based on a standard curve prepared using gallic acid (Sigma-Aldrich, St. Louis, MO, USA).

#### 2.8.2. Total Polysaccharide Content

Total polysaccharide content was analyzed by a method using phenol and sulfuric acid reagents [24]. The extracted ginseng solution (0.6 mL) was mixed with 5% phenol (0.3 mL) using a vortex mixer, and 1.5 mL of 95% (*w*/*w*) concentrated sulfuric acid was added. The mixed solution was shaken for 30 min at 85 °C in a water bath (BF-30SB; Biofree), and cooled for 5 min at room temperature in the dark. Absorbance of the final mixture (0.2 mL) was analyzed at 490 nm using a Multiskan^TM^ GO plate reader (Thermo Scientific). The total polysaccharide content was obtained using a standard curve prepared with d-glucose (Samchun, Seoul, Korea).

#### 2.8.3. Acidic Polysaccharide Content

The acidic polysaccharide content was analyzed using carbazole and sulfuric acid reagents [25]. The extracted ginseng solution (0.5 mL) was mixed with 0.25 mL of carbazole-ethanol reagent (Alfa Aesar, Haverhill, MA, USA), and then with 3 mL of H_2_SO_4_. The mixed solution was shaken for 5 min at 80 °C in a water bath (BF-30SB; Biofree), and cooled for 15 min in the dark at room temperature. The absorbance of the final mixture (0.2 mL) was then analyzed at 525 nm. The acidic polysaccharide content of ginseng was determined using a standard curve prepared with d-galacturonic acid (Sigma, St. Louis, MO, USA).

### 2.9. Ginsenoside Content

After mixing 2 g of ginseng powder with 70% HPLC-grade methanol (25 mL), crude ginsenoside was extracted using a Funnel Shaker (RS-1; Jeio Tech, Dajeon, Korea). The extract was adjusted to 50 mL using a volumetric flask with 70% HPLC-grade methanol. The ginseng suspension was then centrifuged at 3500 rpm for 5 min and filtered with a 0.22 μm syringe filter (Millipore, Burlington, MA, USA). The filtered ginseng solution was subjected to high-performance liquid chromatography (1260 Infinity ll LC System; Agilent, Santa Clara, CA, USA) with a C18 HPLC column (Kinetex, 5 μm, 250 × 4.6 mm; Phenomenex, Seoul, Korea). The temperature condition of the column was set to 30 °C and the flow rate was 1.0 mL/min. The ginsenoside content was measured by solvent gradient analysis with water and acetonitrile, and the content of acetonitrile was set as follows: (0–5 min, 20%; 20 min, 23%; 25 min, 30%; 45 min, 40%; 55–65 min, 50%; and 70–75 min, 20%). The total operating time was 75 min. The injection volume of the sample was 10 μL. The sample absorbance was measured at 203 nm.

### 2.10. Statistical Analysis

All analyses of ginseng powder were conducted at least three times. All result values were indicated as the average value ± standard deviation using the software SPSS 22.0 (SPSS Inc., Chicago, IL, USA). Verification of significant differences was performed using Duncan’s multiple range test (*p* < 0.05) after one-way analysis of variance (ANOVA).

## 3. Results and Discussion

### 3.1. Particle Size

In our preliminary study, the smallest particle size of ginseng was obtained with wet milling and cryogenic milling. The particle size of ginseng powder ground using wet milling (ginseng steeped in H_2_O was homogenized) was 114 μm, based on the D [3,2] value (data not shown). In contrast, the particle size of ginseng powder using cryogenic milling was significantly decreased to 14 μm based on the D [3,2] value (*p* < 0.05). This result is in agreement with a previous study showing that the particle sizes of ginseng roots ground using wet milling and dry milling were 139.3 μm and 15 μm, respectively, under optimum conditions [26]. Therefore, cryogenic milling was used in this study, since particle size reduction was found to be effective. Cryogenic milling is a dry milling process, involving grinding at ultra-low temperatures. It increases the hardness and decreases the extensibility of the ginseng cell wall, thus making it fragile, which results in rapid particle size reduction [18]. In this study, ginseng powders of different sizes were prepared using different conditions of cycle, rate (CPS), and milling time in a cryogenic milling machine (Table 1). Then, the five representative samples (1, 6, 11, 14, and 15) were chosen for each size group from 15 ginseng samples with different sizes, and the chosen samples were roasted for different times. In this study, roasting temperature was 180 °C, which was the optimal temperature (based on the particle size, water solubility, and physiological activity of ginseng) in preliminary experiments conducted at various temperatures (160, 170, 180, 190, and 200 °C). Therefore, roasting was performed for 9, 12, 15, 18, and 21 min according to the roasting time based on 180 °C. Table 2 presents the particle sizes of ginseng powder samples with different roasting times. All roasted treatments demonstrated significant size reduction compared with the control (not roasted), based on the D [3,2] value (*p* < 0.05). This result is in agreement with the previous study, in which roasted ginseng powder (below 30.0 μm) had a smaller particle size than that of non-roasted ginseng powder (1000–1250 μm) [18]. Kim et al. (2004) reported that roasting weakens the starch–protein interactions and destroys the starch granules, eventually reducing the particle size [26]. As the roasting time increased, a decreasing pattern of particle sizes was observed up to 15 min. Im et al. (2010) also showed that the initial particle size (177.2 μm) was decreased with increasing milling times (10, 20, 30, and 40 min), which finally decreased to 92.2 μm after 40 min of grinding [27]. However, when all roasted treatments were roasted for over 18 min, particle size was found to be increased again in this study. These results are consistent with previous studies showing that flocculation of powder occurred as the particle size decreased [28,29]. The flocculation seemed to arise due to van der Waals forces produced by increased surface area and energy [30]. However, the particle size of all roasted ginseng samples was smaller than the control, based on the D [3,2] value (*p* < 0.05). Therefore, roasting seems to be an effective pretreatment for size reduction.

### 3.2. Appearance

**Figure 2:**
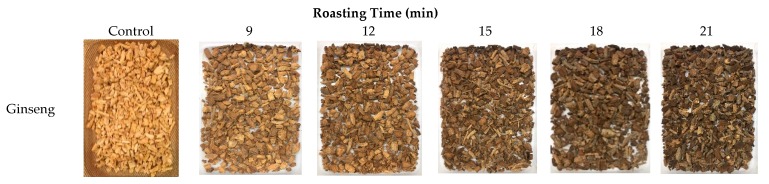
Appearance of dried ginseng root roasted for different times.

**Figure 3:**
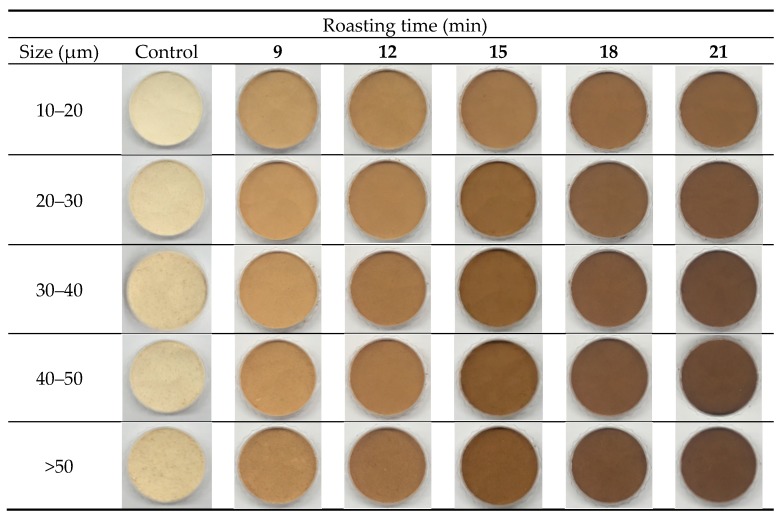
Appearance of dried ginseng root powder pretreated with different roasting times.

The appearances of dried raw ginseng are shown in Figure 2 to compare the changes before and after roasting. As the roasting time increased, the color of ginseng seemed to be gradually changed to black, and started to burn in the case of roasting over 18 min. After dried ginseng was pulverized by cryogenic milling, Figure 3 presents the appearance of ginseng powder before and after roasting. According to the particle sizes, the appearance of the control group (not roasted) was rougher, as the particle size was large. However, the roasted powders seemed to be softer than the control. According to the roasting times, the color of ginseng powders appeared to be gradually darkened with increased roasting time. The color of ginseng powder darkened as the particle size increased and the roasting time increased.

### 3.3. Color

Color changes in ginseng powder were observed according to particle size and roasting time (Table 3). When compared with the control alone, as the particle size increased, the color values showed lower lightness (L*) and higher redness (a*) and yellowness (b*). All color values of controls were significantly different, except for the a* and b^*^ values of the samples with sizes >50 μm (*p* < 0.05). Kim et al. (2012) reported that in general, for powders, L^*^ values increased as the particle size decreased, whereas the a* and b* values varied according to the sample [31]. The smaller the particle size, the higher the scattered light, and thus the higher the L* [21]. With the pretreatment of roasting, L* values of roasted ginseng powders were significantly lower than those of the controls (*p* < 0.05), whereas the a* values were significantly higher than those of the controls (*p* < 0.05). Furthermore, b* values were lower than those of the controls when roasting was done for over 12 min, except for 10–20 μm sized samples (*p* < 0.05). As the roasting time increased in roasted treatments, the L* and b* values of all samples were significantly decreased (*p* < 0.05). Total color difference tended to significantly increase as the roasting time increased (*p* < 0.05). The a* values tended to increase until roasting for 12 min. However, the a^*^ values were significantly decreased when samples were roasted for over 15 min, with the exception of 10–20 μm sized samples (*p* < 0.05). This result was in agreement with a previous study showing that L^*^ and b^*^ values tended to decrease as the roasting time (5.27, 6.19, 6.39, and 8.01 min) increased at 170 °C [32]. Furthermore, the a* values tended to increase at the early stage of roasting and decreased again as the roasting time increased [32]. These color changes were caused by the Maillard reaction of non-enzymatic browning and caramelization [32]. Particle size and roasting time were found to affect the color parameters of ginseng powder.

### 3.4. WSI

**Figure 4:**
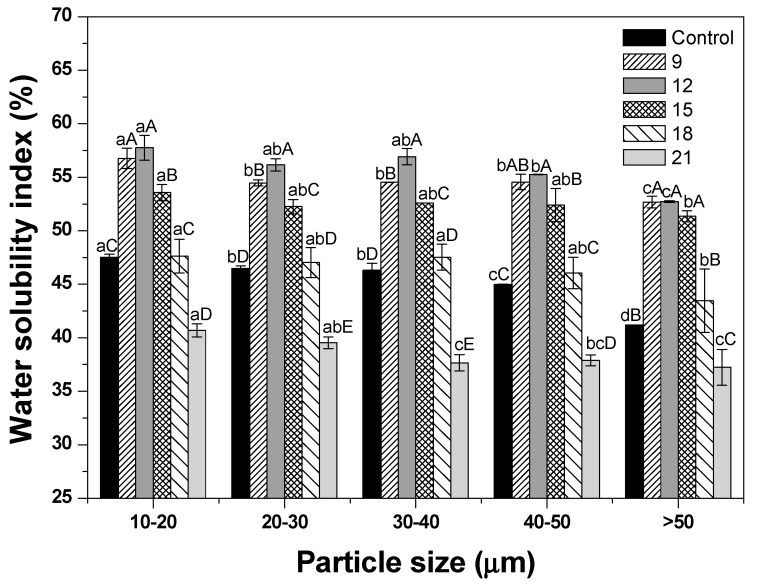
Water solubility index of dried ginseng powders with different particle sizes according to roasting time. After cryogenic milling of ginseng samples with different conditions, the samples were classified by their particle sizes. One treatment condition in each size range was then selected, and samples selected by particle size were then roasted and pulverized by cryogenic milling. ^a–d^ Means with different superscripts within the same time group are significantly different (*p* < 0.05; Duncan’s test). ^A–E^ Means with different superscripts within the same size group are significantly different (*p* < 0.05; Duncan’s test).

Figure 4 presents the WSI of dried ginseng powder with different roasting times. WSI values tended to increase as the particle size became smaller, except for treatments involving roasting at over 18 min. This result was consistent with the findings of a previous study that the WSI value of 30.0 μm-sized ginseng powder was increased by 17% when compared with the 1000–1250 μm-sized ginseng powder [18]. Ultrafine grinding removed the cellulose barrier in the plant material, resulting in a smaller particle size, increased water holding capacity, and ultimately increased WSI [18,33]. In addition to particle size, roasting time also had a significant effect on WSI. WSI values of all treatments except for 18 min and 21 min were higher than those of the control (*p* < 0.05). This result was explained by dextrinization, wherein the starch structure was destroyed by high heat [34]. The starch chain split from amylopectin (insoluble starch) into amylose (soluble starch), thus increasing the amount of soluble material [34]. In addition, another study also reported that glycosidic linkages were broken down at high temperature (approximately 80 °C), resulting in the release of glucose, known as soluble dietary fiber [35]. However, in this study, the WSI values were unexpectedly decreased in the case of roasting over 18 min. Another study has also reported a similar result, showing that when ginseng was roasted for 30 min at 200 °C, the soluble solids content increased until 10 min, but decreased rapidly in cases of roasting for over 10 min [15]. The reason for decreased WSI can be attributed to the burning caused by long heat treatment over 18 min (Figure 2).

### 3.5. Antioxidant Activity

**Figure 5:**
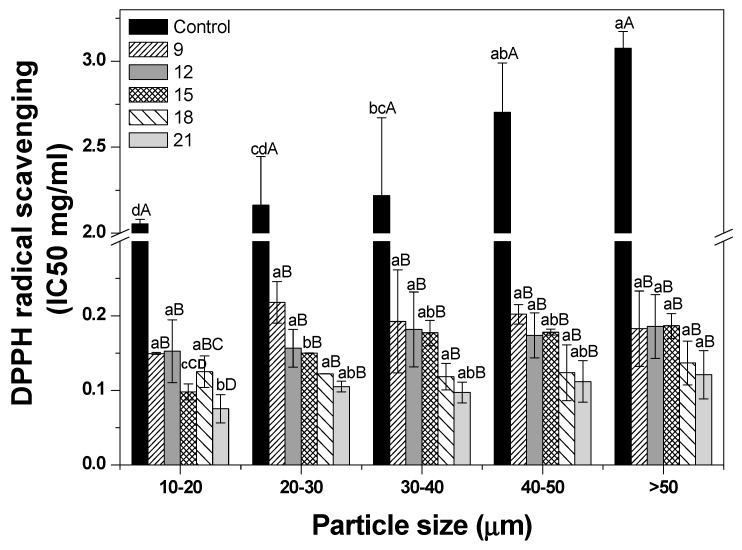
DPPH radical scavenging activity of dried ginseng powders with different particle sizes according to roasting time. After cryogenic milling of ginseng samples with different conditions, the samples were classified by their particle sizes. One treatment condition in each size range was then selected, and samples selected by particle size were then roasted and pulverized by cryogenic milling. ^a–c^ Means with different superscripts within the same time group are significantly different (*p* < 0.05; Duncan’s test). ^A–F^ Means with different superscripts within the same size group are significantly different (*p* < 0.05; Duncan’s test).

The antioxidant activity of ginseng powder is shown in Figure 5. As the particle size decreased, an increasing pattern of antioxidant activity was observed in control samples alone. This result indicates that the ultrafine grinding increased the antioxidant activity by increasing the specific surface area and capillary effect as the particle size decreased (normal grinding, 69.8%; fine grinding, 70.7%; and ultrafine grinding, 83.8%) [36,37,38]. For the roasting effect, the antioxidant activity values of all roasted samples showed no trend according to particle size, whereas that of roasted samples was significantly higher than that of the control (*p* < 0.05). The roasting process appeared to increase melanoidins by reacting reducing sugars and nitrogen oxides through the Maillard reaction, leading to an increase in antioxidant activity [17]. This result is also concordant with the findings of a study by Song et al. (2013) showing that roasting treatment (110 °C, 30 min) resulted in two-times higher antioxidant activity than did the unroasted treatment [39]. Furthermore, antioxidant activity tended to increase with an increase in roasting time, except for the ginseng powder sample sized 10–20 μm. However, it did not show a significant difference. Seong et al. (2018) reported a result consistent with findings of this study and showed an increasing tendency of antioxidant activity as the roasting time increased [40]. However, no significant difference was observed with roasting for 20 and 30 min at 170 °C (*p* > 0.05) [40]. Thus, these results were observed in a narrow range of roasting time in this study.

### 3.6. TPC

**Figure 6:**
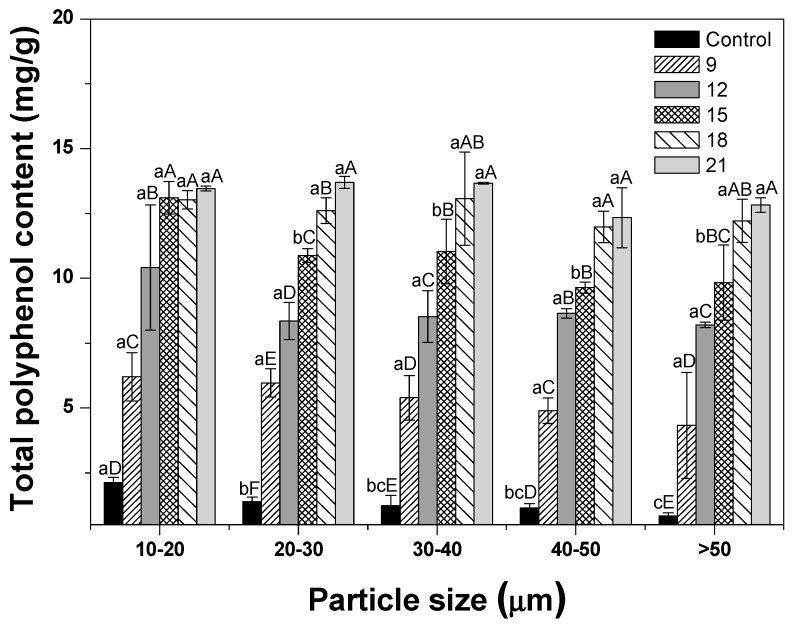
Total polyphenol content of dried ginseng powders with different particle sizes according to roasting time. After cryogenic milling of ginseng samples with different conditions, the samples were classified by their particle sizes. One treatment condition in each size range was then selected, and samples selected by particle size were then roasted and pulverized by cryogenic milling. ^a–c^ Means with different superscripts within the same time group are significantly different (*p* < 0.05; Duncan’s test). ^A–E^ Means with different superscripts within the same size group are significantly different (*p* < 0.05; Duncan’s test).

TPC values of ginseng powder were also analyzed (Figure 6). TPC values showed an increasing pattern when compared with control samples. Furthermore, TPC values of roasted ginseng powder with 10-20 μm-size tended to increase when compared with those of >50 μm samples. These results were also in agreement with the findings of a previous study, in which TPCs displayed an increasing pattern as the particle size decreased in five treatments with various particle sizes (M1, 199.17 μm; M2, 178.27 μm; M3, 85.48 μm; M4. 27.04 μm; and M5 20.97 μm) [41]. This phenomenon was explained by the particle size-extraction relationship, indicating that phenolic compounds were easily released due to an increase in the specific surface area as the particle size decreased [36]. According to the roasting treatment, all roasted treatments showed significantly higher TPC values than the control did (*p* < 0.05). TPC values tended to increase as the roasting time increased. Roasting produced a Maillard reaction product with phenol structure and increased TPC values [42]. This can also be explained by the fact that the treatment changes phenolic acids to a free form, which is easier to release from the food matrix owing to cell disruption [43]. These results were similar to those of a previous study showing that the TPC values of corn kernels increased as the roasting time (10, 20, 30, 40, and 50 min) increased at 180 °C [44].

### 3.7. Polysaccharide Content

**Figure 7:**
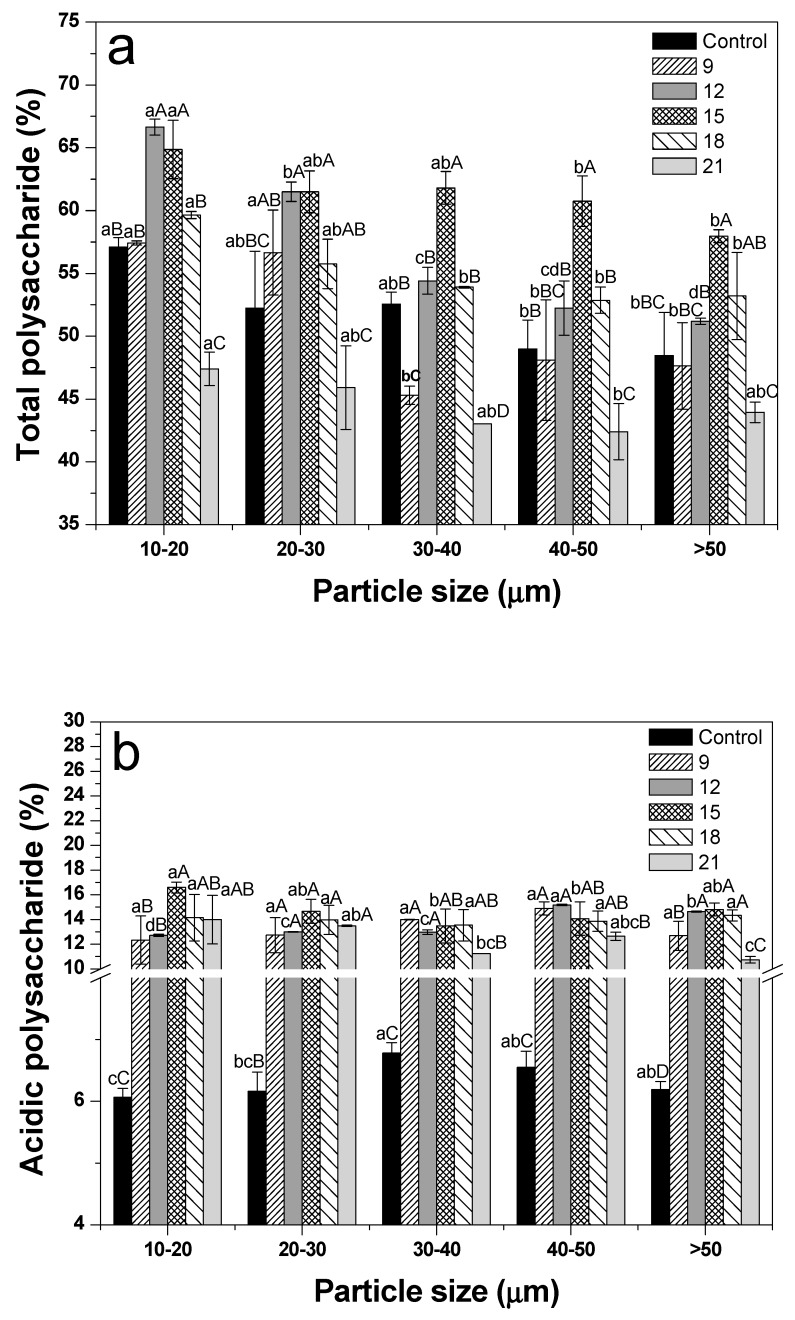
Polysaccharides of dried ginseng powders with different particle size according to roasting time. (**a**) Total polysaccharide; (**b**) acidic polysaccharide. After cryogenic milling of ginseng samples with different conditions, the samples were classified by their particle sizes. One treatment condition in each size range was then selected, and samples selected by particle size were then roasted and pulverized by cryogenic milling. ^a–c^ Means with different superscripts within the same time group are significantly different (*p* < 0.05; Duncan’s test). ^A–E^ Means with different superscripts within the same size group are significantly different (*p* < 0.05; Duncan’s test).

Figure 7a,b present the total and acidic polysaccharide contents, respectively. The total polysaccharide contents of 10–20 μm-sized treatments revealed significantly higher values than the >50 μm-sized treatments, except for the samples roasted for 21 min (*p* < 0.05). Cho et al. (2010) also reported that ginseng samples with sizes smaller than 150 μm had higher total polysaccharide yields compared to samples with higher sizes [45]. However, the acidic polysaccharide contents did not show an increasing pattern as the particle size decreased. This phenomenon appeared to be caused by water holding capacity [46]. Water penetration and adsorption are facilitated by structural changes (specific surface increase, ginseng starch damage) arising from decreasing particle size [46]. As a result, the flocculated powder, due to an increase of the water holding capacity, decreased the diffusion of components from the extractive solvent [46]. For comparison of the roasting treatments, the total polysaccharides of samples sized as 10–20 μm showed the highest value when roasting for 12 min, and the other samples with different sizes showed high contents when roasting for 15 min. However, the total polysaccharides of all treatments tended to gradually decrease with roasting for over 18 min (relatively long heat treatment). In another study, when comparing the light (3–3.5 min), medium (4–5 min), and dark (7–8.5 min) roasting treatments, the total polysaccharide contents of Arabica coffee beans was found to be decreased as the roasting time increased [12]. This was because the long roasting process destroyed or chemically altered the monosaccharides [12]. However, acidic polysaccharides in all roasting treatments showed higher values than the control (not-roasted), despite the absence of any trend according to roasting time (*p* < 0.05). Roasting was considered to decompose the starch components and facilitate the extraction of acidic polysaccharides [47]. This result suggests that the reduction of total polysaccharides (acidic and neutral) is mainly caused by neutral polysaccharide decomposition [48]. Acidic polysaccharides are known to have higher biological activities than neutral polysaccharides in ginseng [48], and thus, roasting can be considered an effective pretreatment.

### 3.8. Ginsenoside Content

Ginsenoside content values showed no tendency of changing according to particle size (Table 4). According to the roasting pretreatment, roasted samples with over 20 μm size had higher contents than the control, except for the samples roasted for 15 min. Seong et al. (2018) reported that the ginsenoside contents of ginseng samples roasted for 20–30 min at 170 °C were higher than those in the control (not roasted) [40]. The ginseng samples with 10–30 μm size revealed the highest ginsenoside contents in the case of roasting for 18 min. However, samples with over 30 μm size indicated the highest contents when roasting for 12 min (*p* < 0.05). These results indicate that the roasting time to obtain the optimum ginsenoside content varied according to particle size.

Rg1, Re, and Rb1, known as the main components of ginseng, also indicated a pattern according to roasting time. Rg1 in all size treatments showed the highest contents with roasting for 9 min (*p* < 0.05). Re showed the highest content with roasting for 12 min, except for samples with 10–20 μm size. Finally, Rb1 of the 10–30 μm-sized samples showed the highest contents with roasting for 18 min, whereas samples with over 30 μm size and relatively larger size showed the highest contents with roasting for 12 min (*p* < 0.05). Therefore, the ginsenoside content was observed to change with particle size and roasting time.

## 4. Conclusions

The purpose of this study was to decrease the particle size of ginseng by roasting and cryogenic milling and eventually increase the water solubility and physiological activity of ginseng.

Ginseng samples were roasted for different times of 9–21 min and ground to different sizes (10–50, and >50 μm) by cryogenic milling. All roasted samples indicated significantly smaller particle sizes compared with the control (not roasted) based on D [3,2] (*p* < 0.05). The particle sizes of the roasted samples showed a decreasing pattern until 15 min of roasting. However, with roasting for over 18 min, the particle size of the ginseng powder was increased due to flocculation. WSI values indicated an increasing pattern as the particle size decreased except for samples roasted at over 18 min. As for the results of antioxidant activity and TPC, samples with 10–20 μm size demonstrated higher values than those for over 50 μm-sized samples. The total polysaccharide contents of 10–20 μm-sized samples revealed significantly higher values than for over 50 μm-sized samples, with an exception of samples roasted for 21 min (*p* < 0.05). However, acidic polysaccharides did not show any trend according to particle size. Ginsenoside contents showed no tendency according to particle size. Based on roasting time, the WSI showed higher values up to 15 min of roasting compared to the control (not roasted) (*p* < 0.05). Antioxidant activity and TPC also showed an increasing trend with increasing roasting time. Total polysaccharides showed the highest values when the samples were roasted for 15 min, except for the particles with 10–20 μm size. Acidic polysaccharide values showed no tendency depending on roasting time, but showed significantly higher values than the control (not roasted). Ginsenoside did not show any pattern depending on roasting time, but the ginsenoside contents of roasted samples with over 20 μm size were higher than the control, except in samples roasted for 15 min. This study proves that roasting and cryogenic milling for certain times have a positive effect, such as reduction of particle size, increased WSI, and increased physiological activity in the manufacture of ginseng powder.

## Figures and Tables

**Table 1:** Treatment of dried ginseng powder by particle size with various cryogenic milling conditions.

		Treatment Conditions			Particle Size (μm)				
Size (μm)	Treatment	Cycle	CPS	Time	D [3,2]	D [4,3]	Dx (10)	Dx (50)	Dx (90)
10–20	1	4	10	4	14.05 ± 0.21 ^jC^	68.24 ± 7.08 ^iB^	5.78 ± 0.15 ^jB^	28.49 ± 0.93 ^gC^	145.88 ± 8.50 ^iB^
	2	4	10	3	14.97 ± 0.70 ^jB^	63.82 ± 4.49 ^iB^	6.13 ± 0.31 ^jA^	30.75 ± 1.24 ^gB^	150.93 ± 8.15 ^iB^
	3	4	10	5	15.52 ± 0.61 ^jA^	89.43 ± 10.93 ^hA^	6.20 ± 0.16 ^jA^	32.15 ± 1.74 ^gA^	193.54 ± 16.51 ^hA^
20–30	4	4	10	2	21.04 ± 2.20 ^iE^	114.43 ± 5.47 ^fgBC^	8.07 ± 0.88 ^iE^	47.55 ± 1.42 ^fC^	308.43 ± 15.75 ^fC^
	5	4	8	3	22.54 ± 0.43 ^hD^	105.13 ± 3.72 ^gC^	8.91 ± 0.26 ^hD^	47.83 ± 0.76 ^fC^	263.75 ± 11.08 ^gD^
	6	4	12	3	24.93 ± 0.35 ^gC^	113.71 ± 4.23 ^fgBC^	9.58 ± 0.13 ^gC^	57.15 ± 0.77 ^eB^	299.17 ± 4.71 ^fCD^
	7	6	15	3	28.74 ± 1.02 ^eA^	131.47 ± 13.30 ^eA^	11.82 ± 0.29 ^eB^	53.69 ± 2.79 ^eA^	377.87 ± 58.51 ^dA^
	8	4	15	3	29.57 ± 0.64 ^fB^	124.00 ± 8.16 ^fB^	13.78 ± 0.15 ^dA^	60.53 ± 2.56 ^eA^	345.50 ± 40.43 ^eB^
30–40	9	8	15	3	31.67 ± 1.16 ^eC^	162.80 ± 17.15 ^eC^	12.92 ± 0.31 ^eB^	60.79 ± 4.72 ^eC^	522.33 ± 54.00 ^dC^
	10	10	15	3	31.90 ± 1.64 ^eC^	177.00 ± 14.38 ^dB^	11.90 ± 0.27 ^fD^	61.05 ± 4.34 ^eC^	567.27 ± 45.37 ^cB^
	11	4	10	1	34.48 ± 0.82 ^dB^	215.55 ± 11.16 ^bA^	12.44 ± 0.28 ^eC^	113.82 ± 9.08 ^bA^	591.73 ± 24.49 ^bcB^
	12	12	15	3	40.02 ± 2.77 ^cA^	226.00 ± 3.00 ^bA^	15.20 ± 0.54 ^cA^	86.65 ± 6.28 ^dB^	664.20 ± 33.04 ^aA^
	13	4	5	3	40.63 ± 1.01 ^cA^	217.63 ± 6.44 ^bA^	15.01 ± 0.33 ^cA^	111.25 ± 4.43 ^bA^	602.38 ± 15.85 ^bB^
40–50	14	2	15	3	46.83 ± 0.99 ^b^	198.80 ± 6.98 ^c^	19.03 ± 0.38 ^b^	104.00 ± 1.41 ^c^	529.40 ± 22.83 ^d^
>50	15	4	10	0.5	64.07 ± 5.31 ^a^	289.00 ± 5.00 ^a^	24.95 ± 2.06 ^a^	207.40 ± 6.27 ^a^	690.40 ± 10.60 ^a^

^a–j^ Means with different superscripts within whole particle size group are significantly different (*p* < 0.05; Duncan’s test). ^A–E^ Means with different superscripts within the similar size group are significantly different (*p* < 0.05; Duncan’s test). Particle size (μm) set up based on D [3,2]. Fifteen treatments were generated according to cycle, CPS (the number of back-and-forth cycles per second), and time conditions, and the larger the number of treatments, the larger the particle size. One cycle is based on pulverization for 3 min at a rate of 15 CPS, oscillating 15 times per second.

**Table 2:** Particle size of dried ginseng powder with various roasting time conditions.

	Roasting ^(1)^	Particle Size (μm)				
Size (μm)	Time (min)	D [3,2]	D [4,3]	Dx (10)	Dx (50)	Dx (90)
10–20	Control	14.00 ± 0.20 ^eA^	68.20 ± 7.10 ^eA^	5.80 ± 0.20 ^eA^	28.50 ± 0.90 ^eA^	145.90 ± 8.50 ^eA^
	9	8.22 ± 0.33 ^eC^	45.78 ± 4.61 ^eB^	3.28 ± 0.11 ^eC^	18.67 ± 1.70 ^eB^	110.00 ± 3.92 ^dB^
	12	7.58 ± 0.27 ^dD^	30.83 ± 2.86 ^eC^	3.04 ± 0.10 ^eD^	15.87 ± 1.13 ^eC^	77.60 ± 8.60 ^dC^
	15	5.60 ± 0.12 ^eF^	20.94 ± 0.88 ^dD^	2.19 ± 0.05 ^eE^	10.29 ± 0.63 ^eE^	55.83 ± 1.18 ^eD^
	18	7.06 ± 0.10 ^eE^	26.03 ± 2.02 ^eCD^	2.90 ± 0.07 ^eD^	13.93 ± 0.30 ^eD^	68.48 ± 6.00 ^dC^
	21	9.27 ± 0.10 ^dB^	29.38 ± 3.99 ^dC^	3.89 ± 0.05 ^dB^	17.77 ± 1.67 ^bB^	70.55 ± 11.40 ^dC^
20–30	Control	24.90 ± 0.40 ^dA^	113.70 ± 4.20 ^dA^	9.60 ± 0.10 ^dA^	57.20 ± 0.80 ^dA^	299.20 ± 4.70 ^dA^
	9	20.34 ± 1.52 ^dB^	112.50 ± 5.45 ^dA^	7.87 ± 0.15 ^dB^	48.94 ± 1.14 ^dB^	288.33 ± 5.03 ^cAB^
	12	16.92 ± 0.63 ^cD^	92.02 ± 13.90 ^dB^	7.10 ± 0.34 ^cC^	35.08 ± 0.30 ^dC^	274.50 ± 19.94 ^cB^
	15	9.80 ± 0.44 ^dE^	52.40 ± 11.92 ^cD^	3.87 ± 0.13 ^dE^	22.56 ± 2.38 ^dD^	118.35 ± 16.78 ^dE^
	18	17.38 ± 0.39 ^dCD^	68.33 ± 5.95 ^dC^	6.51 ± 0.69 ^dD^	35.58 ± 6.31 ^dC^	181.33 ± 14.64 ^cD^
	21	17.62 ± 0.48 ^cC^	97.50 ± 8.48 ^cB^	7.07 ± 0.24 ^cC^	37.62 ± 1.48 ^bC^	252.67 ± 4.16 ^cC^
30–40	Control	34.50 ± 0.80 ^cA^	215.50 ± 11.20 ^bA^	12.40 ± 0.30 ^cA^	113.80 ± 9.10 ^bA^	591.70 ± 24.50 ^bA^
	9	30.60 ± 0.84 ^cB^	181.00 ± 10.86 ^cB^	11.88 ± 0.44 ^cB^	87.08 ± 1.99 ^cB^	503.00 ± 46.90 ^bB^
	12	16.45 ± 0.13 ^cD^	137.40 ± 9.10 ^cD^	6.38 ± 0.12 ^dD^	48.70 ± 1.21 ^cD^	403.17 ± 57.68 ^bC^
	15	12.66 ± 0.59 ^cE^	62.40 ± 2.49 ^cE^	4.92 ± 0.16 ^cE^	31.21 ± 2.28 ^cE^	180.80 ± 6.30 ^cD^
	18	23.58 ± 0.99 ^cC^	143.75 ± 3.86 ^cCD^	9.26 ± 0.48 ^cC^	57.28 ± 2.61 ^cC^	403.20 ± 28.90 ^bC^
	21	24.22 ± 0.79 ^bC^	155.00 ± 3.56 ^bC^	9.27 ± 0.46 ^bcC^	62.08 ± 1.65 ^bC^	445.20 ± 34.80 ^bC^
40–50	Control	46.80 ± 1.00 ^bA^	198.80 ± 7.00 ^cA^	19.00 ± 0.40 ^bA^	104.00 ± 1.40 ^cB^	529.40 ± 22.80 ^cA^
	9	37.98 ± 2.72 ^bB^	208.67 ± 6.81 ^bA^	14.18 ± 1.18 ^bB^	127.25 ± 11.76 ^bA^	524.67 ± 15.50 ^bA^
	12	28.80 ± 0.73 ^bC^	156.60 ± 3.78 ^bB^	11.14 ± 0.46 ^bC^	75.54 ± 1.84 ^bC^	423.80 ± 15.91 ^bB^
	15	14.88 ± 0.53 ^bE^	90.56 ± 11.83 ^bC^	5.98 ± 0.28 ^bE^	41.40 ± 0.88 ^bD^	248.50 ± 29.22 ^bC^
	18	26.83 ± 0.68 ^bD^	156.00 ± 3.87 ^bB^	10.36 ± 0.46 ^bD^	73.58 ± 3.24 ^bC^	427.00 ± 8.03 ^bB^
	21	26.46 ± 0.50 ^bD^	157.40 ± 4.28 ^bB^	10.05 ± 0.32 ^bD^	70.20 ± 1.90 ^bC^	442.00 ± 18.75 ^bB^
>50	Control	64.10 ± 5.30 ^aA^	289.00 ± 5.00 ^aB^	25.00 ± 2.10 ^aA^	207.40 ± 6.30 ^aAB^	690.40 ± 10.60 ^aB^
	9	48.33 ± 0.96 ^aB^	282.25 ± 10.14 ^aB^	18.80 ± 0.75 ^aB^	193.50 ± 7.33 ^aB^	686.00 ± 26.15 ^aB^
	12	45.83 ± 0.51 ^aB^	287.67 ± 0.58 ^aB^	16.53 ± 0.38 ^aB^	197.75 ± 7.14 ^aAB^	708.67 ± 7.77 ^aB^
	15	25.58 ± 0.10 ^aC^	219.40 ± 4.28 ^aC^	9.73 ± 0.12 ^aC^	96.60 ± 3.32 ^aC^	635.20 ± 10.28 ^aC^
	18	43.26 ± 1.31 ^aB^	293.80 ± 14.82 ^aB^	15.76 ± 0.64 ^aB^	192.00 ± 19.7 ^aB^	731.60 ± 19.40 ^aB^
	21	45.96 ± 6.74 ^aB^	338.57 ± 57.82 ^aA^	16.97 ± 3.84 ^aB^	265.71 ± 95.90 ^aA^	781.57 ± 54.01 ^aA^

^a–e^ Means with different superscripts within the same time group are significantly different (*p* < 0.05; Duncan’s test). ^A–F^ Means with different superscripts within the same size group are significantly different (*p* < 0.05; Duncan’s test). ^(^^1)^ After cryogenic milling of ginseng samples with different conditions, the samples were classified with by their particle sizes. One treatment condition in each size range was then selected, and samples selected by particle size were roasted and pulverized by cryogenic milling.

**Table 3:** Color of dried ginseng root powder with various roasting temperature conditions.

	Roasting ^(1)^	Color											
Size (μm)	Time (min)	L*			a*			b*			ΔE		
10–20	Control	82.13	±	0.54 ^aA^	0.71	±	0.22 ^dC^	15.40	±	0.14 ^dC^	-		
	9	65.65	±	0.04 ^aB^	5.26	±	0.00 ^eB^	17.03	±	0.04 ^cA^	17.18	±	0.02 ^dE^
	12	57.65	±	0.05 ^aC^	6.01	±	0.02 ^eA^	16.16	±	0.03 ^aB^	25.06	±	0.05 ^dD^
	15	52.35	±	0.08 ^aD^	5.44	±	0.03 ^dB^	14.32	±	0.06 ^aD^	30.17	±	0.08 ^eC^
	18	45.95	±	0.07 ^aE^	5.87	±	0.01 ^aA^	12.36	±	0.01 ^aE^	36.67	±	0.07 ^eB^
	21	44.53	±	0.05 ^aF^	5.42	±	0.01 ^aB^	11.50	±	0.03 ^aF^	38.11	±	0.03 ^dA^
20–30	Control	80.44	±	0.25 ^bA^	1.07	±	0.04 ^cF^	16.03	±	0.36 ^cB^	-		
	9	60.99	±	0.06 ^cB^	5.94	±	0.01 ^dC^	17.02	±	0.03 ^cA^	20.08	±	0.06 ^aE^
	12	54.27	±	0.13 ^bC^	6.29	±	0.01 ^dA^	15.53	±	0.03 ^bC^	26.65	±	0.10 ^cD^
	15	44.59	±	0.09 ^bD^	6.04	±	0.05 ^bB^	11.90	±	0.03 ^cD^	36.42	±	0.09 ^aC^
	18	44.03	±	0.02 ^bE^	5.55	±	0.02 ^cD^	11.26	±	0.01 ^bE^	37.00	±	0.02 ^dB^
	21	40.31	±	0.01 ^bF^	5.27	±	0.00 ^bE^	9.57	±	0.00 ^bF^	40.86	±	0.01 ^bA^
30–40	Control	78.77	±	0.77 ^cA^	1.41	±	0.09 ^bF^	16.64	±	0.26 ^bB^	-		
	9	61.23	±	0.05 ^bB^	6.01	±	0.01 ^cC^	17.24	±	0.01 ^aA^	18.15	±	0.05 ^cE^
	12	51.71	±	0.14 ^cC^	6.66	±	0.02 ^bA^	15.08	±	0.03 ^cC^	27.61	±	0.14 ^bD^
	15	43.83	±	0.16 ^cD^	6.32	±	0.02 ^aB^	12.28	±	0.02 ^bD^	35.51	±	0.14 ^bC^
	18	42.03	±	0.02 ^cE^	5.71	±	0.02 ^bD^	10.60	±	0.02 ^cE^	37.48	±	0.02 ^bB^
	21	38.19	±	0.13 ^cF^	4.99	±	0.01 ^cE^	8.22	±	0.05 ^cF^	41.60	±	0.12 ^aA^
40–50	Control	77.57	±	0.04 ^dA^	1.83	±	0.02 ^aF^	17.21	±	0.01 ^aA^	-		
	9	59.63	±	0.08 ^dB^	6.49	±	0.01 ^bB^	17.18	±	0.05 ^bA^	18.54	±	0.08 ^bE^
	12	51.65	±	0.14 ^cC^	6.56	±	0.03 ^cA^	14.77	±	0.00 ^dB^	26.56	±	0.04 ^cD^
	15	43.36	±	0.03 ^dD^	5.91	±	0.03 ^cC^	11.23	±	0.05 ^dC^	34.97	±	0.03 ^dC^
	18	40.78	±	0.14 ^dE^	5.54	±	0.02 ^cD^	9.81	±	0.04 ^dD^	37.71	±	0.13 ^dB^
	21	37.72	±	0.07 ^dF^	4.99	±	0.02 ^cE^	8.01	±	0.01 ^dE^	41.00	±	0.06 ^bA^
>50	Control	76.31	±	0.10 ^eA^	1.91	±	0.05 ^aE^	17.26	±	0.01 ^aA^	-		
	9	56.92	±	0.08 ^eB^	6.83	±	0.03 ^aA^	16.80	±	0.05 ^dB^	20.01	±	0.08 ^aE^
	12	49.10	±	0.12 ^eC^	6.86	±	0.00 ^aA^	14.04	±	0.01 ^eC^	27.85	±	0.13 ^aD^
	15	41.77	±	0.06 ^eD^	5.95	±	0.04 ^cB^	10.84	±	0.03 ^eD^	35.36	±	0.06 ^cC^
	18	40.08	±	0.06 ^eE^	5.34	±	0.00 ^dC^	9.22	±	0.02 ^eE^	37.28	±	0.05 ^cB^
	21	37.35	±	0.18 ^eF^	4.97	±	0.01 ^dD^	7.90	±	0.02 ^eF^	40.18	±	0.17 ^cA^

^a–e^ Means with different superscripts within the same time group are significantly different (*p* < 0.05; Duncan’s test). ^A–F^ Means with different superscripts within the same size group are significantly different (*p* < 0.05; Duncan’s test). ^(^^1)^ After cryogenic milling of ginseng samples with different conditions, the samples were classified by their particle sizes. One treatment condition in each size range was then selected, and samples selected by particle size were roasted and pulverized by cryogenic milling.

**Table 4:** Ginsenosides content of dried ginseng powder with different roasting time conditions.

	Roasting ^(1)^	Ginsenosides Content (mg/g)							
**Size (μm)**	**Time (min)**	**Rg1**	**Re**	**Rf**	**Rb1**	**Rc**	**Rb2**	**Rb3**	**Rd**
10–20	Control	3.68 ± 0.03 ^C^	3.41 ± 0.02 ^D^	1.46 ± 0.08 ^B^	4.26 ± 0.03 ^C^	1.14 ± 0.04 ^C^	0.97 ± 0.03 ^C^	0.19 ± 0.02 ^C^	0.31 ± 0.01 ^C^
	9	4.62 ± 0.02 ^A^	2.82 ± 0.01 ^E^	1.01 ± 0.01 ^C^	4.18 ± 0.04 ^C^	1.01 ± 0.03 ^D^	0.91 ± 0.01 ^C^	0.15 ± 0.00 ^C^	0.35 ± 0.00 ^C^
	12	3.91 ± 0.13 ^B^	7.56 ± 0.21 ^B^	1.77 ± 0.00 ^A^	8.34 ± 0.30 ^B^	3.35 ± 0.13 ^B^	2.99 ± 0.11 ^B^	0.51 ± 0.02 ^B^	0.91 ± 0.03 ^B^
	15	2.30 ± 0.14 ^E^	1.84 ± 0.10 ^F^	0.73 ± 0.01 ^D^	3.04 ± 0.03 ^D^	1.05 ± 0.03 ^CD^	0.92 ± 0.01 ^C^	0.17 ± 0.01 ^C^	0.18 ± 0.01 ^D^
	18	4.00 ± 0.09 ^B^	8.01 ± 0.17 ^A^	1.85 ± 0.04 ^A^	9.88 ± 0.82 ^A^	4.07 ± 0.10 ^A^	3.60 ± 0.11 ^A^	0.66 ± 0.03 ^A^	1.02 ± 0.05 ^A^
	21	3.35 ± 0.05 ^D^	6.88 ± 0.14 ^C^	0.27 ± 0.08 ^E^	ND ^E^	ND ^E^	ND ^D^	ND ^D^	0.21 ± 0.02 ^D^
	**Time (min)**	**Rg2(S)**	**Rg2(R)**	**Rg3(S)**	**Rg3(R)**	**Rh1(S)**	**Rh2(S)**	**Sum**	
	Control	0.17 ± 0.01 ^D^	0.09 ± 0.01 ^C^	ND ^B,(2)^	ND ^B^	0.10 ± 0.02 ^B^	ND	15.77 ± 0.02 ^bC^	
	9	0.15 ± 0.01 ^D^	0.14 ± 0.00 ^BC^	ND ^B^	ND ^B^	0.03 ± 0.00 ^D^	ND	15.39 ± 0.09 ^bC^	
	12	0.73 ± 0.02 ^B^	0.48 ± 0.02 ^A^	ND ^B^	ND ^B^	0.04 ± 0.00 ^D^	ND	30.64 ± 1.03 ^cB^	
	15	0.26 ± 0.04 ^C^	0.10 ± 0.04 ^C^	0.19 ± 0.01 ^A^	0.07 ± 0.03 ^A^	0.22 ± 0.00 ^A^	ND	11.06 ± 0.03 ^cD^	
	18	0.89 ± 0.02 ^A^	0.34 ± 0.30 ^AB^	ND ^B^	ND ^B^	0.07 ± 0.00 ^C^	ND	34.39 ± 1.10 ^aA^	
	21	ND^E^	0.19 ± 0.04 ^BC^	ND ^B^	ND ^B^	ND^E^	ND	10.73 ± 0.00 ^dD^	
**Size (μm)**	**Time (min)**	**Rg1**	**Re**	**Rf**	**Rb1**	**Rc**	**Rb2**	**Rb3**	**Rd**
20–30	Control	3.28 ± 0.04 ^B^	2.68 ± 0.02 ^C^	0.81 ± 0.70 ^B^	3.60 ± 0.06 ^D^	0.90 ± 0.02 ^D^	0.78 ± 0.02 ^D^	0.19 ± 0.01 ^C^	0.28 ± 0.02 ^D^
	9	4.59 ± 0.07 ^A^	2.37 ± 0.03 ^CD^	0.96 ± 0.03 ^B^	4.24 ± 0.06 ^C^	1.12 ± 0.01 ^C^	1.03 ± 0.01 ^D^	0.17 ± 0.00 ^C^	0.38 ± 0.01 ^C^
	12	3.10 ± 0.05 ^B^	8.88 ± 0.15 ^A^	1.65 ± 0.06 ^A^	9.62 ± 0.15 ^A^	4.14 ± 0.06 ^A^	3.64 ± 0.07 ^A^	0.62 ± 0.01 ^A^	1.14 ± 0.01 ^A^
	15	2.17 ± 0.07 ^C^	2.08 ± 0.08 ^D^	0.73 ± 0.03 ^B^	3.08 ± 0.16 ^E^	1.05 ± 0.00 ^C^	0.84 ± 0.02 ^D^	0.14 ± 0.01 ^C^	0.23 ± 0.02 ^E^
	18	2.98 ± 0.37 ^B^	8.71 ± 0.24 ^A^	1.67 ± 0.17 ^A^	9.71 ± 0.34 ^A^	4.09 ± 0.10 ^A^	3.29 ± 0.41 ^B^	0.60 ± 0.09 ^A^	1.11 ± 0.02 ^A^
	21	2.11 ± 0.03 ^C^	5.47 ± 0.35 ^B^	1.60 ± 0.10 A	6.49 ± 0.04 B	3.14 ± 0.01 ^B^	2.88 ± 0.00 ^C^	0.51 ± 0.02 ^B^	0.87 ± 0.05 ^B^
	**Time (min)**	**Rg2(S)**	**Rg2(R)**	**Rg3(S)**	**Rg3(R)**	**Rh1(S)**	**Rh2(S)**	**Sum**	
	Control	0.06 ± 0.02 ^E^	0.06 ± 0.01 ^E^	ND ^C^	ND ^B^	0.10 ± 0.01 ^C^	ND	12.75 ± 0.07 ^cD^	
	9	0.15 ± 0.00 ^D^	0.14 ± 0.00 ^D^	ND ^C^	ND ^B^	0.04 ± 0.00 ^E^	ND	15.18 ± 0.20 ^bC^	
	12	0.93 ± 0.01 ^B^	0.63 ± 0.01 ^A^	ND ^C^	ND ^B^	0.04 ± 0.00 ^E^	ND	34.39 ± 0.55 ^bA^	
	15	0.32 ± 0.02 ^C^	0.16 ± 0.01 ^D^	0.19 ± 0.02 ^B^	0.05 ± 0.01 ^A^	0.22 ± 0.03 ^B^	ND	11.26 ± 0.03 ^bcE^	
	18	0.97 ± 0.02 ^B^	0.56 ± 0.07 ^B^	ND ^C^	ND ^B^	0.07 ± 0.01 ^D^	ND	34.41 ± 0.97 ^aA^	
	21	1.21 ± 0.08 ^A^	0.39 ± 0.03 ^C^	0.46 ± 0.03 ^A^	ND ^B^	0.26 ± 0.02 ^A^	ND	25.80 ± 0.15 ^aB^	
**Size (μm)**	**Time (min)**	**Rg1**	**Re**	**Rf**	**Rb1**	**Rc**	**Rb2**	**Rb3**	**Rd**
30–40	Control	3.93 ± 0.08 ^B^	3.24 ± 0.04 ^D^	1.13 ± 0.31 ^C^	4.60 ± 0.28 ^D^	1.57 ± 0.16 ^D^	1.33 ± 0.17 ^C^	0.19 ± 0.04 ^D^	0.42 ± 0.07 ^E^
	9	5.20 ± 0.08 ^A^	2.94 ± 0.03 ^E^	1.04 ± 0.01 ^C^	5.16 ± 0.09 ^C^	1.42 ± 0.03 ^D^	1.29 ± 0.04 ^C^	0.21 ± 0.01 ^D^	0.49 ± 0.01 ^D^
	12	3.20 ± 0.02 ^C^	9.94 ± 0.03 ^A^	1.75 ± 0.07 ^A^	11.14 ± 0.02 ^A^	4.54 ± 0.06 ^A^	4.00 ± 0.04 ^A^	0.68 ± 0.01 ^B^	1.30 ± 0.01 ^A^
	15	2.14 ± 0.05 ^F^	2.26 ± 0.07 ^F^	0.68 ± 0.00 ^D^	3.39 ± 0.07 ^E^	1.26 ± 0.09 ^E^	1.06 ± 0.06 ^D^	0.15 ± 0.04 ^E^	0.26 ± 0.03 ^F^
	18	3.02 ± 0.10 ^D^	8.59 ± 0.22 ^B^	1.81 ± 0.06 ^A^	10.01 ± 0.37 ^B^	4.31 ± 0.13 ^B^	4.03 ± 0.11 ^A^	0.74 ± 0.03 ^A^	1.14 ± 0.02 ^B^
	21	1.79 ± 0.02 ^E^	4.70 ± 0.08 ^C^	1.51 ± 0.03 ^B^	5.40 ± 0.06 ^C^	2.53 ± 0.05 ^C^	2.29 ± 0.03 ^B^	0.43 ± 0.00 ^C^	0.74 ± 0.02 ^C^
	**Time (min)**	**Rg2(S)**	**Rg2(R)**	**Rg3(S)**	**Rg3(R)**	**Rh1(S)**	**Rh2(S)**	**Sum**	
	Control	0.20 ± 0.08 ^E^	0.11 ± 0.12 ^E^	ND ^C^	ND ^B^	0.05 ± 0.09 ^C^	ND	16.77 ± 0.10 ^aE^	
	9	0.20 ± 0.00 ^E^	0.20 ± 0.00 ^D^	ND ^C^	ND ^B^	0.05 ± 0.00 ^C^	ND	18.20 ± 0.25 ^aD^	
	12	1.04 ± 0.01 ^C^	0.73 ± 0.01 ^A^	ND ^C^	ND ^B^	0.05 ± 0.00 ^C^	ND	38.36 ± 0.21 ^aA^	
	15	0.32 ± 0.01 ^D^	0.09 ± 0.00 ^E^	0.18 ± 0. 00 ^B^	0.05 ± 0.00 ^A^	0.20 ± 0.04 ^B^	ND	12.05 ± 0.03 ^aF^	
	18	1.13 ± 0.04 ^B^	0.56 ± 0.01 ^B^	0.02 ± 0.01 ^C^	ND^B^	0.10 ± 0.01 ^C^	ND	35.46 ± 1.01 ^aB^	
	21	1.28 ± 0.03 ^A^	0.38 ± 0.01 ^C^	0.67 ± 0.01 ^A^	ND^B^	0.33 ± 0.01 ^A^	ND	22.05 ± 0.31 ^cC^	
**Size (μm)**	**Time (min)**	**Rg1**	**Re**	**Rf**	**Rb1**	**Rc**	**Rb2**	**Rb3**	**Rd**
40–50	Control	3.14 ± 0.02 ^C^	2.70 ± 0.01 ^E^	1.05 ± 0.03 ^C^	3.23 ± 0.14 ^E^	0.73 ± 0.06 ^E^	0.66 ± 0.04 ^F^	0.17 ± 0.01 ^C^	0.25 ± 0.02 ^E^
	9	4.93 ± 0.03 ^A^	3.41 ± 0.05 ^D^	1.08 ± 0.01 ^C^	5.38 ± 0.06 ^D^	1.35 ± 0.02 ^C^	1.20 ± 0.05 ^D^	0.19 ± 0.01 ^C^	0.49 ± 0.01 ^D^
	12	3.67 ± 0.06 ^B^	8.68 ± 0.11 ^A^	1.61 ± 0.04 ^B^	10.22 ± 0.14 ^A^	4.01 ± 0.05 ^A^	3.55 ± 0.06 ^A^	0.58 ± 0.00 ^A^	1.23 ± 0.04 ^A^
	15	2.16 ± 0.03 ^F^	1.85 ± 0.01 ^F^	0.76 ± 0.01 ^D^	2.95 ± 0.05 ^F^	1.04 ± 0.06 ^D^	0.91 ± 0.03 ^E^	0.18 ± 0.07 ^C^	0.19 ± 0.01 ^F^
	18	2.94 ± 0.02 ^D^	8.46 ± 0.06 ^B^	1.78 ± 0.02 ^A^	9.53 ± 0.16 ^B^	4.03 ± 0.02 ^A^	3.44 ± 0.03 ^B^	0.62 ± 0.01 ^A^	1.08 ± 0.02 ^B^
	21	2.36 ± 0.03 ^E^	5.67 ± 0.06 ^C^	1.64 ± 0.02 ^B^	6.93 ± 0.06 ^C^	2.63 ± 0.02 ^B^	2.29 ± 0.06 ^C^	0.45 ± 0.01 ^B^	0.72 ± 0.03 ^C^
	**Time (min)**	**Rg2(S)**	**Rg2(R)**	**Rg3(S)**	**Rg3(R)**	**Rh1(S)**	**Rh2(S)**	**Sum**	
	Control	0.07 ± 0.01 ^E^	0.02 ± 0.01 ^E^	ND ^D^	ND ^B^	0.11 ± 0.01 ^B^	ND	12.13 ± 0.03 ^cE^	
	9	0.21 ± 0.00 ^D^	0.25 ± 0.00 ^C^	ND ^D^	ND ^B^	0.05 ± 0.00 ^C^	ND	18.52 ± 0.22 ^aD^	
	12	0.86 ± 0.01 ^C^	0.67 ± 0.01 ^A^	ND ^D^	ND ^B^	0.05 ± 0.00 ^C^	ND	35.14 ± 0.50 ^bA^	
	15	0.27 ± 0.08 ^D^	0.16 ± 0.04 ^D^	0.19 ± 0.02 ^B^	0.05 ± 0.05 ^A^	0.23 ± 0.06 ^A^	ND	10.93 ± 0.04 ^cF^	
	18	1.12 ± 0.03 ^B^	0.65 ± 0.01 ^A^	0.03 ± 0.00 ^C^	ND ^B^	0.12 ± 0.00 ^B^	ND	33.73 ± 0.42 ^aB^	
	21	1.19 ± 0.01 ^A^	0.47 ± 0.01 ^B^	0.38 ± 0.01 ^A^	ND ^B^	0.25 ± 0.00 ^A^	ND	24.99 ± 0.28 ^bC^	
**Size (μm)**	**Time (min)**	**Rg1**	**Re**	**Rf**	**Rb1**	**Rc**	**Rb2**	**Rb3**	**Rd**
>50	Control	3.81 ± 0.10 ^B^	3.27 ± 0.05 ^D^	1.50 ± 0.05 ^B^	4.20 ± 0.11 ^E^	1.10 ± 0.05 ^E^	0.94 ± 0.05 ^E^	0.20 ± 0.03 ^C^	0.29 ± 0.03 ^E^
	9	4.47 ± 0.16 ^A^	3.53 ± 0.08 ^D^	1.13 ± 0.04 ^C^	5.33 ± 0.19 ^D^	1.53 ± 0.06 ^D^	1.36 ± 0.05 ^D^	0.24 ± 0.01 ^C^	0.47 ± 0.01 ^D^
	12	2.91 ± 0.07 ^C^	8.32 ± 0.32 ^A^	1.52 ± 0.03 ^B^	9.65 ± 0.33 ^A^	4.15 ± 0.18 ^A^	3.77 ± 0.15 ^A^	0.62 ± 0.03 ^A^	1.18 ± 0.07 ^A^
	15	2.22 ± 0.01 ^E^	1.98 ± 0.05 ^E^	0.81 ± 0.00 ^D^	3.14 ± 0.03 ^F^	1.14 ± 0.07 ^E^	0.96 ± 0.03 ^E^	0.20 ± 0.06 ^C^	0.27 ± 0.03 ^E^
	18	2.74 ± 0.03 ^D^	7.55 ± 0.09 ^B^	1.66 ± 0.02 ^A^	8.91 ± 0.05 ^B^	3.73 ± 0.02 ^B^	3.27 ± 0.03 ^B^	0.62 ± 0.01 ^A^	0.97 ± 0.02 ^B^
	21	1.96 ± 0.06 ^F^	4.76 ± 0.22 ^C^	1.50 ± 0.03 ^B^	5.86 ± 0.17 ^C^	2.48 ± 0.06 ^C^	2.22 ± 0.05 ^C^	0.42 ± 0.00 ^B^	0.72 ± 0.02 ^C^
	**Time (min)**	**Rg2(S)**	**Rg2(R)**	**Rg3(S)**	**Rg3(R)**	**Rh1(S)**	**Rh2(S)**	**Sum**	
	Control	0.16 ± 0.03 ^E^	0.09 ± 0.01 ^F^	ND ^D^	ND ^D^	0.11 ± 0.02 ^C^	ND	15.66 ± 0.04 ^bE^	
	9	0.29 ± 0.02 ^D^	0.25 ± 0.01 ^D^	ND ^D^	ND ^D^	0.05 ± 0.00 ^D^	ND	18.66 ± 0.62 ^aD^	
	12	0.91 ± 0.03 ^C^	0.55 ± 0.01 ^B^	0.01 ± 0.02 ^D^	ND ^D^	0.04 ± 0.00 ^D^	ND	33.64 ± 1.22 ^bA^	
	15	0.33 ± 0.04 ^D^	0.17 ± 0.02 ^E^	0.26 ± 0.02 ^B^	0.12 ± 0.00 ^A^	0.21 ± 0.00 ^B^	ND	11.82 ± 0.02 ^abF^	
	18	1.09 ± 0.00 ^B^	0.57 ± 0.00 ^A^	0.06 ± 0.00 ^C^	0.06 ± 0.00 ^B^	0.11 ± 0.00 ^C^	ND	31.36 ± 0.22 ^bB^	
	21	1.21 ± 0.03 ^A^	0.40 ± 0.01 ^C^	0.50 ± 0.01 ^A^	0.03 ± 0.02 ^C^	0.27 ± 0.02 ^A^	ND	22.32 ± 0.61 ^cC^	

^a–c^ Means with different superscripts within the same time group are significantly different (*p* < 0.05; Duncan’s test). ^A–F^ Means with different superscripts within the same size group are significantly different (*p* < 0.05; Duncan’s test). ^(1)^ After cryogenic milling of ginseng samples with different conditions, the samples were classified by their particle sizes. One treatment condition in each size range was then selected, and samples selected by particle size were roasted and pulverized by cryogenic milling. ^(^^2)^ ND stands for not detected.

## References

[1] Yun T.K. (2001). Brief introduction of *Panax ginseng* CA Meyer. J. Korean Med. Sci..

[2] Park S.Y., Lee K.Y., Cho Y.J., Park B.K., Kim K.J., Lee N.R., Kim D.G., Kim Y.H., Hahn T.W. (2015). Efficacy of orally administered ginseng stem and leaf in chickens. Korean J. Vet. Res..

[3] Li X.G. (1992). Studies on the transforming mechanism of amino acid components in ginseng in the course of ginseng process. J. Ginseng Res..

[4] Kang K.S., Yokozawa T., Yamabe N., Kim H.Y., Park J.H. (2007). ESR study on the structure and hydroxyl radical-scavenging activity relationships of ginsenosides isolate from *Panax* ginseng CA Meyer. Biol. Pharm. Bull..

[5] Park C.K., Jeon B.S., Yang J.W. (2003). The chemical components of Korean ginseng. Food Ind. Nutr..

[6] Lee J.H., Choi K.H., Sohn E.H., Jang K.H. (2013). Quality characteristics and ginsenosides composition of ginseng-*Yakju* according to the particle size of ginseng powder. Nutr. Food Sci. Prev..

[7] Choi K.T. (2008). Botanical characteristics, pharmacological effects and medicinal compo-nents of Korean *Panax ginseng* CA Meyer. Acta Pharmacol. Sin..

[8] Lu J.M., Yao Q., Chen C. (2009). Ginseng compounds: An update on their molecular mechanisms and medical applications. Curr. Vasc. Pharmacol..

[9] Choi K.O., Lee I., Park S.Y.R., Kim D.E., Lim J.D., Kang W.S., Ko S.H. (2012). Ultrafine *Angelica gigas* powder normalizes ovarian hormone levels and has antiosteoporosis properties in ovariectomized rats: Particle size effect. J. Med. Food.

[10] Suh C.S., Chun J.K. (1981). Relationships among the roasting conditions, colors and extractable solid content of roasted barley. Korean J. Food Sci. Technol..

[11] Redgwell R.J., Trovato V., Curti D. (2003). Cocoa bean carbohydrates: Roasting induced changes and polymer interactions. Food Chem..

[12] Redgwell R.J., Trovato V., Curti D., Fischer M. (2002). Effect of roasting on degradation and structural features of polysaccharides in Arabica coffee beans. Carbohydr. Res..

[13] Saklara S., Unganb S., Katnasc S. (2003). Microstructural changes in hazelnuts during roasting. Food Res. Int..

[14] Jeon E.J., Kim K.Y., Lee J.E., Catherine W., Kwon J.H. (2008). Monitoring of Roasting Conditions for the Functional Properties of Lateral Root of Red Ginseng. Korean J. Food Preserv..

[15] Park M.H., Kim K.C. (1995). Changes in Physicochemical Components of Ginseng Marc by Roasting Process. Korean J. Ginseng Sci..

[16] Sivetz M., Desrosier N.W. (1979). Coffee technology. United States Department of Agriculture National Agricultural Library.

[17] Yoon S.K., Kim W.J. (1989). Effects of roasting conditions on quality and yields of barley tea. Korean J. Food Sci. Technol..

[18] Lee B.G., Lee K.Y., Jorge S., Jorge R., Baek H., Min J.H., Kang W.S. Ultrafine powderization using low temperature turbo mill to improve water solubility of red ginseng powder. Proceedings of the 2012 12th IEEE International Conference on Nanotechnology (IEEE-NANO).

[19] Manohar B., Sridhar B.S. (2001). Size and shape characterization of conventionally and cryogenically ground turmeric (*Curcuma domestica*) particles. Powder Technol..

[20] Meghwal M., Goswami T.K. (2013). Evaluation of size reduction and power requirement in ambient and cryogenically ground fenugreek powder. Adv. Powder Technol..

[21] Lee S.B., Yoo S.H., Ganesan P., Kwak H.S. (2013). Physicochemical and antioxidative properties of Korean nanopowdered white ginseng. Food Sci. Technol..

[22] Blois M.S. (1958). Antioxidant determinations by the use of a stable free radical. Nature.

[23] Ough C.S., Amerine M.A. (1988). Methods for Analysis of Musts and Wine.

[24] Dubois M., Gillers K.A., Hamilton J.K., Rebers P.A., Smith J. (1956). Colorimetric method for determination of sugar and related substance. Anal. Chem..

[25] Do J.H., Lee H.O., Lee S.K., Jang J.K., Lee S.D., Sung H.S. (1993). Colorimetric Determination of Acidic Polysaccharide from *Panax ginseng*, its Extraction Condition and Stability. J. Ginseng Res..

[26] Kim W., Choi S., Kerr W., Johnson J., Gaines C. (2004). Effect of heating temperature on particle size distribution in hard and soft wheat flour. J. Cereal Sci..

[27] Im G.Y., Jang S.Y., Jeong Y.J. (2010). Quality characteristics of *Panax ginseng* CA Meyer with steaming heat and wet grinding conditions. J. Korean Soc. Food Sci. Nutr..

[28] Lee J.S., Lee H.S. (2007). Effect of grinding methods on particle size and crystalline structure of copper phthalocyanine. J. Ind. Eng. Chem..

[29] Jo G.S., Sin J.S., Kim J.H. (2004). Measurement of particle size and particle size distribution. Polym. Sci. Technol..

[30] Uhm Y.R., Kim J.W., Jung J.W., Rhee C.K. (2009). The fabrication of PVA polymer coated on the surface of B_4_C nanocomposite by high energy ball mill. J. Korean Powder Metall..

[31] Kim H.Y., Seo H.I., Ko J.Y., Kim J.I., Lee J.S., Song S.B., Jung T.W., Kim K.Y., Kwak D.Y., Oh I.S. (2012). Physicochemical characteristics of the sorghum (*Sorghum bicolor* L. Moench) powder following low temperature-microparticulation. Koran J. Food Nutr..

[32] Kim E.K., Jeong Y.H., Gu S.Y., Song K.Y., Kim I.Y., Kim K.Y. (2019). Physicochemical characteristics of Brazilian coffea arabica cv. Catuai coffee extracts with different roasting conditions. J. Korean Soc. Food Sci. Nutr..

[33] Hemansson A. (1982). Gel characteristics-structure as related to texture and water binding of blood plasma gels. J. Food Sci..

[34] Guha M., Ali S.Z., Bhattacharya S. (1997). Twin-screw extrusion of rice flour without a die: Effect of barrel temperature and screw speed on extrusion and extrudate characteristics. J. Food Eng..

[35] Zavareze E.D.R., Dias A.R.G. (2011). Impact of heat-moisture treatment and annealing in starches: A review. Carbohydr..

[36] Cho Y.J. (2014). Antioxidant, angiotensinconverting enzyme and xanthin oxidase inhibitory activity of extracts from *Saururus chinensis* leaves by ultrafine grinding. Korean J. Food Preserv..

[37] Kim K.H., Lee I.H., Lee H.S., Park J.K. (2013). R & D trend and information analysis of nanoparticles. Pros. Ind. Chem..

[38] Kim C.S., Kim H.I. (2009). Physicochemical properties of non-waxy rice flour affected by grinding methods and steeping times. J. Korean Soc. Food Sci. Nutr..

[39] Song Y.B., Lee K.S., Lee M.S., Kim A.J. (2013). Bioactivity changes in mung beans according to the roasting time. Korean J. Food Nutr..

[40] Seong B.J., Kim S.I., Jee M.G., Kim S.D., Kwon A.R., Kim H.H., Hwang Y.G., Lee K.S. (2018). Physicochemical characteristics according to the roasting conditions and grinding grade for the development of drip type red ginseng. J. Korean Soc. Food Sci. Nutr..

[41] Cha S.M., Son B.Y., Lee J.S., Baek S.B., Kim S.L., Ku J.H., Hwang J.J., Song B.H., Woo S.H., Kwon Y.U. (2012). Effect of particle size on physico-chemical properties and antioxidant activity of corn silk powder. Korean J. Crop. Sci..

[42] Durmaz G., Alpaslan M. (2007). Antioxidant properties of roasted apricot (*Prunus armeniaca* L.) Kernel. Food Chem..

[43] Kim J.S., Kang O.J., Gweon O.C. (2013). Comparison of phenolic acids and flavonoids in black garlic at different thermal processing steps. J. Funct. Foods.

[44] Chung H.S., Chung S.K., Youn K.S. (2011). Effects of roasting temperature and time on bulk density, soluble solids, browning index and phenolic compounds of corn kernels. J. Food Process Pres..

[45] Cho K.L., Woo H.J., Lee I.S., Lee J.W., Cho Y.C., Lee I.N., Chae H.J. (2010). Optimization of Enzymatic Pretreatment for the Production of Fermented Ginseng using Leaves, Stems and Roots of Ginseng. J. Ginseng Res..

[46] Cho C.W., Kim S.W., Rho J.H., Rhee Y.K. (2008). Extraction characteristics of saponin and acidic polysaccharide based on the red ginseng particle size. J. Ginseng Res..

[47] Lee J.W., Do J.H. (2002). Extraction condition of acidic polysaccharide from Korean red ginseng marc. J. Ginseng Res..

[48] Zhang X., Yu L., Bi H., Li X., Ni W., Han H., Li N., Wang B., Zhou Y., Tai G. (2009). Total fractionation and characterization of the water soluble polysaccharides iso-lated from panax ginsen C.A. Meyer. Carbohydr. Polym..

